# A statistical model to identify hereditary and epigenetic fusion genes associated with dilated cardiomyopathy

**DOI:** 10.3389/fgene.2024.1438887

**Published:** 2024-10-01

**Authors:** Ling Fei, Jun Zhang, Degen Zhuo

**Affiliations:** ^1^ Department of Cardiology, Chengdu Xinhua Hospital, Tianjin Medical University, Tianjin, China; ^2^ Department of Cardiology, Changzhou Central Hospital, Tianjin Medical University, Cangzhou, Hebei, China; ^3^ SplicingCodes, BioTailor Inc, Miami, FL, United States

**Keywords:** hereditary, germline, fusion gene, dilated cardiomyopathy, epigenetic. RNA-Seq, inheritance, genomics

## Abstract

Dilated cardiomyopathy (DCM) is a heart condition that causes enlarged and weakened left ventricles and affects the heart’s ability to pump blood effectively. Most genetic etiology still needs to be understood. Previously, we have used the known germline hereditary fusion genes (HFGs) to identify HFGs associated with multiple myeloma and leukemia. In this study, we have developed a statistical model to study fusion transcripts discovered from the left ventricles of 122 DCM patients and 252 GTEx (Genotype Tissue Expression) healthy controls to discover novel HFGs, ranging from 4% to 87.7%, and EFGs, ranging from 4% to 99.2%, associated with DCM. This discovery of numerous novel HFGs and EFGs associated with DCM provides first-hand evidence that DCM results from interactive developmental consequences between germline genetic and environmental abnormalities and paves the way for future research and diagnostic and therapeutic applications, instilling hope for the future of DCM treatment.

## Introduction

Dilated cardiomyopathy (DCM) is a heart disease characterized by the enlarged and thin muscle walls of one or both ventricles. The thinner (dilated) walls are weak and cannot pump blood adequately to the rest of the body. DCM is the most common indication for heart transplantation and the third most common cause of heart failure (Maron et al., 2006). The prevalence of DCM is estimated to be one out of 2,500 people in the general population (Hershberger et al., 2013; Weintraub et al., 2017). However, this estimation may be significantly underestimated (Diamant et al., 2003). The Global Burden of Disease study showed that the total global prevalence of cardiomyopathy was about 2.5 million cases (Schultheiss et al., 2019; Tayal et al., 2021; Harding et al., 2023). The DCM disease disproportionately affects men more frequently than women, and outcomes are worse in patients of Black African descent (Ingle, 2008; Dries et al., 1999). The DCM onset occurs between 20 and 60 years old, although DCM can affect children and constitute 60% of cardiomyopathy (Billings et al., 2018). Electrocardiogram, chest X-ray, and echocardiogram are used to diagnose DCM.

The causes of DCM can be classified as genetic and acquired, though the two are not mutually exclusive. The pathogenesis of DCM patients is quite heterogeneous due to the broad spectrum of genetic and environmental abnormalities leading to the appearance of the phenotype of the disorder (Roberts et al., 2015; Haggerty et al., 2019). Studies from the 1990s established that idiopathic DCM (IDC) frequently has a genetic etiology and is considered familial when more than one first-degree relative has been diagnosed with DCM or has experienced a sudden cardiac death (SCD) (Mestroni et al., 1999; Lewsey and Breathett, 2021). About 25%–35% of affected individuals have familial forms of DCM, with most cases inherited in an autosomal dominant form (Chen et al., 2021). Recent comprehensive targeted sequencing studies showed that 40%–50% of these families have an identifiable monogenic cause (Hershberger et al., 2013), or at least a rare variant of large effect size as the primary determinant of risk (Pugh et al., 2014). More than twenty mutated genes have been identified as the causative genes for familial DCM, including *TTN*, *LMNA*, *FLNC*, *SCN5A*, *JPH2*, *PLN*, *RYR2*, *DSP*, *RBM2*), and *BAG3* (Tayal et al., 2021; Harding et al., 2023; Chen et al., 2021; Yamada and Nomura, 2021). The primary causative genes for DCM are *TTN* (Gerull et al., 2002; Norton et al., 2013; Herman et al., 2012) and *LMNA* (Zahr and Jaalouk, 2018; Brodt et al., 2013; Levine et al., 2016). Recent advances in RNA-Seq and next-generation sequencing (NGS) have made it possible for scientists to study the gene expression of DCM (Alimadadi et al., 2020; Yuan et al., 2022; Huang et al., 2019; Januel et al., 2021).

The fusion gene is a hybrid gene formed from two previously independent genes of the reference genome. It has been thought to result from random somatic genomic abnormalities and is associated with cancer. Unlike *BCR-ABL1* generated by somatic genomic alterations, germline structural variants have been the foundation of germline HFGs. Congenital red-green color blindness is an inherited disease caused by the germline structural variants (Hayashi et al., 1999; Nathans et al., 1986). Investigations have shown that congenital red-green color blindness is caused by germline chimeric genes of *OPN1LW* and *OPN1MW* genes at position Xq28 (Hayashi et al., 1999; Drummond-Borg et al., 1989). Many inherited peripheral neuropathies are caused by *CMT1A* gene duplication. α-thalassemia is an inherited blood disorder that is shown to be associated with deletions/inactivation of chromosome 16p (Farashi and Harteveld, 2018). Smith-Magenis syndrome (SMS) is a developmental disorder affecting behavior, emotions, and learning processes and has been shown to be caused by a ∼4-Mb heterozygous interstitial deletion on chromosome 17p11.2 in ∼80%–90% of affected patients (Bi et al., 2003). These discoveries have been performed by traditional molecular technologies, which have often been costly, time-consuming, and labor-intensive. More recently, analyses of whole-genomic and RNA-Seq sequencing of five generations show that germline chimeric *KANK1-DMRT1* transcript derived from a complex structural variant is associated with a congenital heart defect (da Costa et al., 2024). Complex genomic rearrangements cause rare diseases (Schuy et al., 2022). More recently, Raghav et al. analyzed RNA-Seq data fromTarget ALS and the NYGC ALS Consortium and identified 607 unique “gene fusions,” some significantly higher in ASL samples than those in control (Raghav et al., 2024). However, the keys to successful analyses of whole-genomic and RNA-Seq data depend on the accurate identifications of fusion junctions of fusion transcripts and structural variants.

To accurately identify fusion transcripts and minimize potential artifacts of fusion transcripts, we used the splicing code theory to develop Splicing code Identify Fusion Transcripts (*SCIF*) to discover fusion transcripts at the maximum accuracy (Zhou et al., 2017). During experimental validation of fusion transcripts, we observed that fusion transcripts were present in healthy tissues at such high frequencies that it was mathematically impossible to generate them from somatic abnormalities (Zhou et al., 2017). We systematically validated *KANSARL* (*KANSL1-ARL17A*) as the first predisposition (germline) fusion gene specific to 29% of the population of European ancestry (Zhou et al., 2017). *TPM4-KLF2*, detected in 92.2% of 727 multiple myeloma patients, was also positive in all five healthy controls (Fei et al., 2022). These widespread presences of fusion transcripts in healthy populations systematically forced us to study hereditary fusion genes (HFGs). We have defined HFGs as the fusion genes offspring inherited from their parents, excluding epigenetic (readthrough) fusion transcripts generated by *cis*-splicing of readthrough pre-mRNAs of two identical neighboring genes (Zhuo, 2022). Using monozygotic (MZ) twins as a genetic model, we identified 1,180 hereditary fusion genes from 37 pairs of monozygotic twins (Zhuo, 2022). We used 1,180 HFGs discovered in monozygotic twins to analyze fusion transcripts from 390 acute myeloid leukemia (AML) patients and identified 242 hereditary fusion genes ranging from 10% to 82.2% and were associated with AML (Ling and Zhuo, 2022). Similarly, hereditary fusion genes are associated with amyotrophic lateral sclerosis (ALS) (Yang et al., 2023). In this study, we develop a robust statistics model to perform comparative statistical analysis between dilated cardiomyopathy patients and GTEx healthy controls to discover hereditary and epigenetic fusion genes associated with non-cancerous dilated cardiomyopathy (DCM).

## Materials and methods

### Materials

#### Dilated cardiomyopathy (DCM)RNA-Seq dataset

DCM RNA-Seq data (Accession: PRJEB8360) were downloaded from NCBI (https://www.ncbi.nlm.nih.gov/bioproject/PRJEB8360/). RNA-Seq dataset contained 366 RNA-Seq data samples from left ventricle heart tissues of 122 dilated cardiomyopathy (DCM) of 128 subjects with end-stage heart failure. According to the data description, all subjects were transported to the Royal Brompton and Harefield NHS Foundation Trust (London, United Kingdom). In the final analyzed dataset, we parsed 366 RNA-Seq data from 122 DCM patients out of 384 RNA-Seq data from 128 DCM patients.

#### Get left ventricle heart RNA-Seq data

The Genotype-Tissue Expression (GTEx) RNA-Seq data (dB Gap: phs000424) was downloaded from NCBI (https://www.ncbi.nlm.nih.gov/bioproject/PRJNA75899). We selected and performed RNA-Seq data of left ventricle heart tissues of 252 GTEx healthy controls with unique IDs. We used them as health controls to investigate whether fusion genes are associated with DCM.

### Methods

#### Identification of total fusion transcripts from RNA-Seq data

We used SCIF (SplicingCodes Identify Fusion Genes v.1.0) to perform RNA-Seq analysis of 366 DCM RNA-Seq samples and 252 RNA-Seq samples at the default conditions described previously (Zhou et al., 2017). The detailed steps to produce SCIF and parameters can be found in the Supplementary Material of the paper (Zhou et al., 2017).

#### Discovery of hereditary (germline) fusion genes generated by genomic abnormalities

Since HFGs were defined as the fusion genes offspring inherited from their parents, excluding epigenetic (readthrough) fusion transcripts generated by *cis*-splicing of readthrough pre-mRNAs of two identical-strand neighboring genes (Zhuo, 2022). Genomic abnormalities generating fusion genes included inversion, gene duplications, insertion, deletion, and translocations (intra-chromosomal or inter-chromosomal). For fusion genes generated by insertions, deletions, and intra-chromosomal translocations, if the intergenic gaps of two parental genes are larger than 200,000 bp, they were associated with genomic abnormalities. If offspring inherit fusion genes from their parents, they are germline hereditary fusion genes (HFGs).

#### Discovery of epigenetic fusion genes (EFGs)

To distinguish fusion genes generated by genomic alterations, we defined epigenetic fusion genes as fusion transcripts generated by *cis*-splicing of readthrough pre-mRNAs of two identical-strand neighboring genes (Zhuo, 2022). If intergenic gaps between two identical strand neighboring genes were equal to and smaller than 200,000 bp, the fusion transcripts generated by these two genes were epigenetic fusion genes. Therefore, all human populations have potentially identical EFGs.

#### A statistical model to identify hereditary and epigenetic fusion genes

To develop a robust statistical model to identify hereditary and epigenetic fusion genes, we first used a well-documented germline *KANSARL* (*KANSL1-ARL17A*) fusion gene specific to the population of European ancestry origin but absent from those of Asia and Africa as a reference. We used three sets of statistical parameters to analyze and compare RNA-Seq data from Asia, Africa, Europe, and North America to identify consistent parameters of producing expected outcomes. Then, we used all humans with almost identical sets of epigenetic fusion genes (EFGs) to validate if the parameters used in the analysis could obtain consistent results. When setting α = 0.01 and α/2 = 0.005,we can obtain Z_α/2_ = 2.576; we found that n*p ≥ 5 enable us to obtain hereditary and epigenetic fusion genes, which are statistically significant, where n and p are sample size and percentage of samples with a specific positive fusion gene. Then, we classified fusion transcripts into HFGs and EFGs based on the definitions described above and performed downstream analyses. Then, we analyzed and summarized the HFG and EFG data independently.

#### A simple validation algorithm of the hereditary and epigenetic fusion gene data

To validate the hereditary and epigenetic fusion gene data in this study, we can use the fusion junction sequences provided in Supplementary Tables S1, S2 to obtain the results in this study. The following algorithm is able to generate results identical to those presented in this paper except for the fusion genes generated via highly repetitive genomic duplications.

1. Download RNA-Seq data from NCBI.
2. Use the fusion junction sequences to produce a hash table.
3. Read in each RNA-Seq read and scan the hash table to see if the fusion junction sequences are present.
4. Count, analyze, and compare isoforms of thehereditary and epigenetic fusion genes.

The algorithm described above also allows scientists and readers to discover the hereditary and epigenetic fusion genes associated with other diseases and complex traits. BLAST can be used to search NCBI databases to determine whether the fusion genes are from highly repetitive sequences.

## Results

### Identification of fusion transcripts from RNA-Seq data of dilated cardiomyopathy (DCM) patients

**FIGURE 1:**
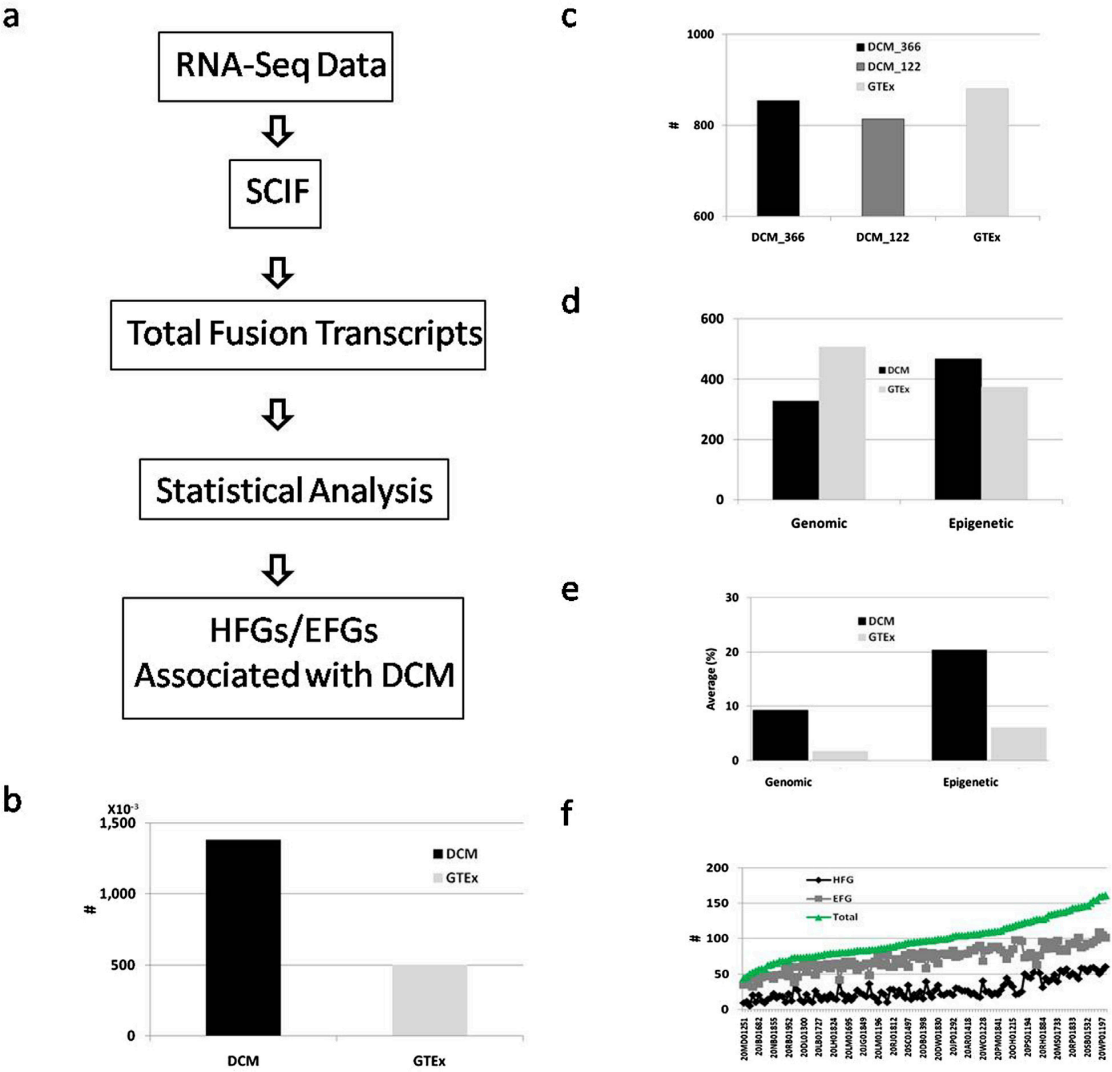
Identification and analysis of fusion transcripts from DCM and GTEx. **(A)**. Schematic diagram of a simplified procedure for identifying HFG and EFG fusion transcripts associated with DCM. The solid black and light gray rectangles represented the total fusion transcripts of the DCM patients and GTEx healthy controls; **(B)**. Comparison of the total fusion transcripts between DCM and GTEx; **(C)**. Comparison of numbers of fusion transcripts identified in ≥5 DCM RNA-Seq SRA samples, ≥5 DCM patients, and ≥5 GTEx healthy controls. The solid black, dark gray, and light gray rectangles showed the numbers of fusion transcripts of ≥5 DCM RNA-Seq samples, ≥5 unique DCM patients, and ≥5 GTEx healthy controls, respectively; **(D)**. Classification and comparisons of genomic and epigenetic fusion transcripts of ≥5 unique DCM patients and ≥5 GTEx healthy controls. The solid black and gray rectangles displayed the numbers of fusion transcripts of ≥5 unique DCM patients and ≥5 GTEx healthy controls; **(E)**. Comparisons of average frequencies of fusion transcripts of ≥5 DCM patients and ≥5 GTEx healthy controls. The solid black and gray rectangles showed the fusion transcripts of ≥5 unique DCM patients and ≥5 GTEx healthy controls; **(F)**. The HFG, EFG, and total transcripts distribution among 122 DCM patients. The black, gray, and green lines represented the HFG, EFG, and total transcripts, respectively.

To discover germline hereditary fusion genes (HFGs) associated with dilated cardiomyopathy (DCM), we downloaded RNA-Seq data from 384 SRA (Sequence Read Archive) experiments, which were generated by Max Delbrück Center for Molecular Medicine, Berlin-Buch, Germany, and from left ventricle heart tissue of 128 dilated cardiomyopathy (DCM) cases from NCBI (Accession: PRJEB8360). According to the information, the cardiac samples were collected when transplants were performed for all DCM patients at the Royal Brompton and Harefield NHS Foundation Trust. From 384 RNA-Seq samples of 128 DCM patients, we obtained analytic results of 366 RNA-Seq samples of 122 DCM patients. We downloaded RNA-Seq data of left ventricle heart tissues of 252 GTEx healthy controls with unique IDs used as healthy controls to investigate whether fusion genes are associated with DCM. As shown in Figure 1A, we first used SCIF (SplicingCodes Identify Fusion Transcripts) to discover total fusion transcripts from DCM patients and GTEx healthy controls at default conditions. We identified 138,000 fusion transcripts from 122 DCM patients, the average of which was 1,131 fusion transcripts of three RNA-Seq data per patient (Figure 1B).

There were 855 and 814 fusion transcripts of ≥5 SRA samples and ≥ five unique DCM patients, counting only 0.62% and 0.59% of the total DCM fusion transcripts. The slight differences between fusion transcripts with ≥5 RNA-Seq SRA samples and with ≥5 unique DCM patients suggested that increased numbers of RNA-Seq SRA experiments did not significantly affect the total numbers of fusion transcripts with recurrent frequencies of ≥5 RNA-Seq SRA samples (Figure 1C). In comparison, we discovered 49,900 fusion transcripts from 252 GTEx healthy controls (Figure 1B), the average of which was 198 fusion transcripts per GTEx healthy control. We identified 881 fusion transcripts with recurrent frequencies of ≥5 GTEx healthy controls, counting only 1.8% of 49,900 GTEx fusion transcripts. Exceptionally low percentages of ≥5 GTEx and DCM samples suggested that highly recurrent fusion transcripts were rare, not randomly distributed, and potentially associated with the intrinsic characteristics of the datasets.

To characterize fusion transcripts more accurately, we classified 814 DCM fusion transcripts of ≥5 DCM patients into two groups: fusion transcripts generated by genomic abnormalities and epigenetic fusion transcripts generated via *cis-*splicing of readthrough pre-mRNAs. We identified 328 transcripts generated by genomic alterations and 486 epigenetic fusion transcripts (Figure 1D). In comparison, we classified 881 fusion transcripts of ≥5 GTEx healthy controls into 507 fusion transcripts generated by genomic alterations and 374 epigenetic fusion transcripts. Figure 1E showed that the frequency average of DCM fusion transcripts formed by genomic alterations was 9.3%, while its GTEx counterpart was 1.7%. The former was 5.5-fold of the latter. The average frequency of DCM epigenetic fusion transcripts was 20.4%. Its GTEx counterpart was 6.1% (Figure 1E). The former was 3.3-fold of the latter, suggesting that epigenetic fusion transcripts increased 3.3-fold during DCM development. Figure 1F shows that the average frequency of GTEx epigenetic fusion transcripts was 3.6-fold of the GTEx genomic fusion transcripts, confirming that genomic fusion transcripts differed from epigenetic fusion transcripts.

Next, we examined the distribution of fusion transcripts of ≥5 DCM patients among different DCM patients. Figure 1E shows that 122 DCM patients ranged from 44 to 161 fusion transcripts per DCM patient, the average of which was 99.2, while 252 GTEx healthy controls ranged from 1 to 123 fusion transcripts per GTEx healthy control, the average of which was 49.7. Even though the total DCM fusion transcripts were 5.7-fold of the GTEx counterpart, the former was only 2-fold of the latter, indicating that additional RNA-Seq numbers dramatically expanded collections of DCM fusion transcripts expressed at low levels.

We examined fusion transcripts with ≥5 DCM patients to investigate distributions of these fusion transcripts among different individuals. Figure 1F shows that 328 fusion genes generated by genomic alterations were distributed among 122 DCM patients and ranged from 5 to 64 per DCM patient, the average of which was 27.8. Figure 1F shows that the 486 epigenetic fusion transcripts were apportioned among 122 DCM patients and ranged from 32 to 109 per DCM patient, the average of which was 71.3. The epigenetic average was 2.6-fold that of the genomic counterpart, suggesting that DCM patients had an increased expression of epigenetic fusion genes associated with DCM. The more fusion transcripts generated by DCM patients’ genomic alterations, the more epigenetic fusion transcripts the DCM patients had (Figure 1F), suggesting that epigenetic fusion transcripts reflected genetic and environmental alterations.

### Develop a statistical model to discover hereditary and epigenetic fusion genes associated with dilated cardiomyopathy (DCM)

As described above, fusion genes generated by genomic alterations were detected at high frequencies in both DCM patients and GTEx healthy controls. One of the natural questions was whether these fusion genes resulted from germline structural variants or somatic genomic rearrangements. Since structural variants generating a fusion gene per individual had a rate of 3.6 × 10^−2^ (Conrad et al., 2010b; Campbell and Eichler, 2013), the possibility of generating ≥5 identical fusion transcripts by random genomic alteration was 6 × 10^−8^. The probability of identical fusion transcripts per individual was 7.3 × 10^−6^ in DCM patients and 1.5 × 10^-5^in GTEx healthy controls. Due to DCM patients and GTEx healthy controls being a no-cancerous disease, the possibilities of 7.3 × 10^−6^ and 1.5 × 10^−5^ were little chance and mathematically unlikely for these fusion transcripts to be generated by somatic genomic abnormalities. Hence, we could conclude that fusion transcripts with ≥5 DCM and GTEx healthy controls generated by genomic alterations were from germline genomic structural variants or inherited from their parents. Previously, we defined the hereditary fusion genes (HFGs) as fusion genes offspring inherited from their parents, excluding epigenetic fusion genes, defined as the fusion genes generated via *cis-*splicing of readthrough pre-mRNAs of two identical strand neighboring genes. Therefore, these DCM fusion transcripts generated by genomic alterations were treated as hereditary fusion genes (HFGs). Instead of using the known HFGs to discover HFGs associated with human disease, we used Z-tests to compare DCM fusion transcripts with GTEx fusion transcripts and set Z_*α/2*_ equal to 2.576. Supplementary Table S1 showed that 210 HFGs encoding 224 fusion transcripts were identified to be associated with DCM, which was statistically significant. Among 210 HFGs associated with DCM, 206 HFGs coding for 220 HFG transcripts were positively associated with DCM. Only four HFGs were negatively associated with DCM and had preventive effects on DCM. 220 HFG transcripts positively associated with DCM ranged from 4.1% to 87.7%, with an average of 9.1%. In comparison, we detected these HFG counterparts in 0%–22.2% of 252 GTEx healthy controls, the average of which was 1.12%. The former average was 8.2-fold of the GTEx healthy control counterpart. 8.2-fold differences between DCM and GTEx and only four HFGs negatively associated with DCM suggested that these HFGs dramatically increased DCM.

Similarly, we used the statistical method to analyze EFGs of DCM patients and GTEx healthy controls. We discovered 181 epigenetic fusion genes (EFGs) coding for 217 EFG transcripts ranging from 4.1% to 99.2%, the average of which was 20.4% (Supplementary Table S2). In comparison, their GTEx counterparts ranged from zero to 81%, the average of which was 6.1%. The average frequency of EFG transcripts associated with DCM was 3.3-fold that of the GTEx counterpart, suggesting that environmental abnormalities were essential for DCM. Only *SIDT2-TAGLN* EFG was negatively associated with DCM, and 216 EFG transcripts were positively associated with DCM, implying that most genetic and environmental abnormalities promoted DC initiation, development, and prognosis. If the genetic and environmental abnormalities leading to DCM counted for 100%, the ratio of differences between DCM and GTEx HFG frequency averages vs. their EFG counterpart was 8.1:3.3 and equal to 2.48:1. Therefore, we obtained germline HFG abnormalities which contributed 71.3% of DCM. In comparison, environmental abnormalities contributed to 28.7% of DCM. This data suggested that genetic abnormalities represented by HFGs were much more powerful driving forces than environmental ones.

### Characterize germline HFGs associated with DCM

**FIGURE 2:**
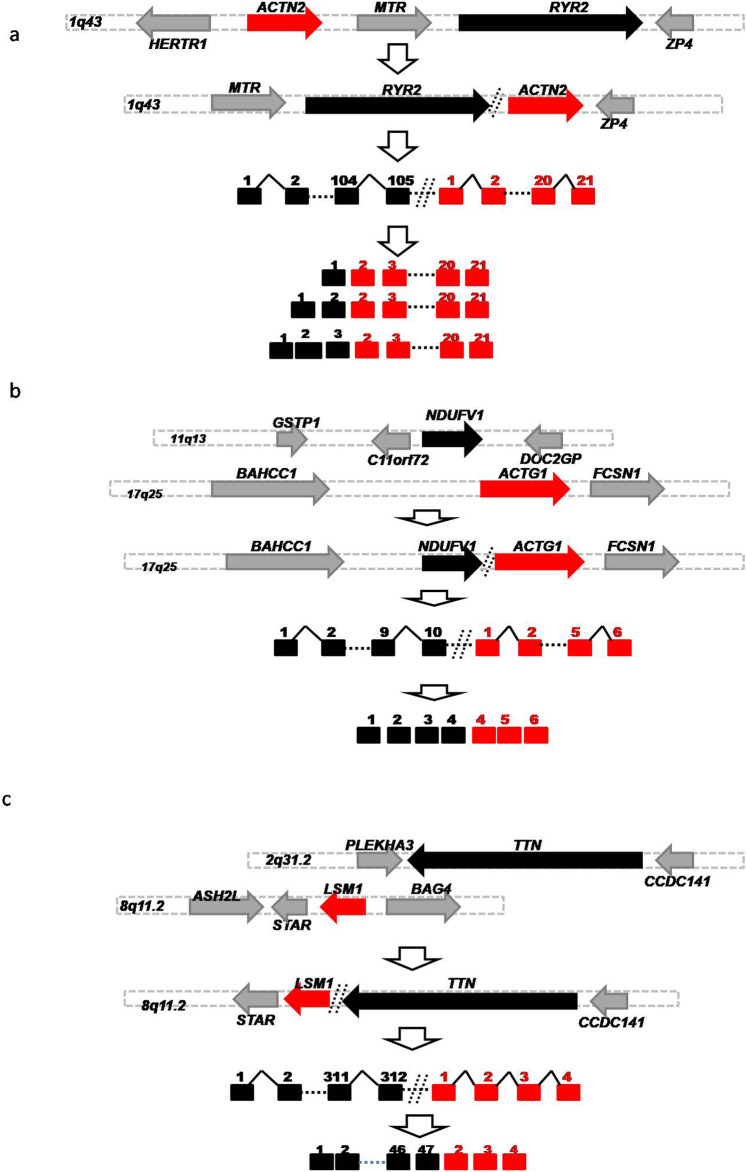
Schematic diagrams of Germline HFGs’ association with DCM. Schematic diagrams showed potential germline genomic abnormalities to generate *RYR2-ACTN2.* **(A)** *NDUFV1-ACTG1* **(B)** and *TTN-LSM1* **(C)**. The solid black, red, and gray horizontal arrows represented the five ‘genes, 3'genes, and genes surrounded by 5′ and 3′ genes. The solid black and red squares were 5′ and 3′exons. The solid black angle line and dashed line were introns and omitted sequences. Open vertical arrows indicated potential steps from genomic alterations to producing fusion gene sequences. The numbers above the squares were exon numbers.

To characterize these HFGs, we examined 206 HFGs coding for 220 HFG transcripts positively associated with DCM. Table 1 shows the top 11 HFGs encoding 12 HFG transcripts positively associated with DCM, which ranged from 20% to 87.7%. They ranged from 1.2% to 22.2% of GTEx healthy controls. The most recurrent HFG was *RYR2-ACTN2*, detected in 87.7% of left ventricle heart tissues of 122 DCM patients and 21.4% of 252 GTEx counterparts (Table 1). The DCM *RYR2-ACTN2* recurrent frequency was four-fold that of the GTEx counterpart. As shown in Table 1, the second *RYR2-ACTN2* isoform was detected in 22.1% of DCM patients and 1.2% of GTEx healthy controls. The DCM frequency of the second *RYR2-ACTN2* isoform was 18-fold of the GTEx counterpart and suggested that increasing *RYR2-ACTN2* expression and/or *RYR2-ACTN2* alternative splicing significantly increased possibilities of DCM. Figure 2A showed that *RYR2* and *ACTN2* were located on 1q43 and encoded ryanodine receptor 2 (cardiac) and actinin α2. A potential inversion of *ACTN2→RYR2* structure resulted in *RYR2→ACTN2* structure, and the first *RYR2* exon replaced the first *ACTN2* exon to form putative 867 aa (cardiac) ryanodine receptor 2-actinin α2 fusion protein, which was 27 aa shorter than actinin α2. *RYR2-ACTN2* HFG expressed twenty-five isoforms in DCM patients. In comparison, only eight *RYR2-ACTN2* isoforms were detected in GTEx healthy controls. Figure 2A shows that three *RYR2-ACTN2* main isoforms were detected in 87.7%, 22.1%, and 14.8% of 122 DCM patients, while their GTEx counterparts were observed in 21.4%, 1.2%, and 0% of 252 GTEx healthy controls. The former were four-, 18-, and 37-fold of the latter, suggesting that increasing *RYR2-ACTN2* gene expression and/or *RYR2-ACTN2* alternative splicing were associated with DCM initiation, development, and prognosis.

**TABLE 1:** The highly-recurrent germline hereditary fusion genes (HFGs) associated with DCM.

HFGs	DCM (122)		GTEx (252)		Folds
	#	%	#	%	
*RYR2-ACTN2*	107	87.70	54	21.43	4.1
*NDUFV1-ACTG1*	89	72.95	45	17.86	4.1
*TINAGL1-ACTG1*	62	50.82	56	22.22	2.3
*TTN-LSM1*	49	40.16	19	7.54	5.3
*C20orf166-C9orf3*	48	39.34	23	9.13	4.3
*C20orf166-MYH6*	45	36.89	42	16.67	2.2
*PCBP3-USP28*	39	31.97	12	4.76	6.7
*MYH7-C9orf3*	31	25.41	10	3.97	6.4
*TPM2-ACTG1*	30	24.59	30	11.90	2.1
*TTN-LMF1*	29	23.77	7	2.78	8.6
*RYR2-ACTN2*	27	22.13	3	1.19	18.6
*TTN-PPFIA1*	25	20.49	13	5.16	4.0

After examining HFGs associated with DCM, we found that multiple different 5′genes were fused with a single 3′gene. Supplementary Table S3 showed that twenty-five different 5′genes were fused with 3′- *ACTG1* genes encoding actin gamma 1 to form different 3′-*ACTG1-*fused HFGs. Supplementary Table S3 showed that the frequencies of 3′-*ACTG1*-fused HFGs were observed in 4.1% to 73% of DCM patients, while they were detected in 0% to 22.2% of GTEx healthy controls. The former were 2.1- to 24.8 folds of the latter. Consequently, these large numbers of different 3′-*ACTG1* fused HFGs produced diverse ACTG1 fusion proteins and provided diverse transcription regulation disrupting 3′-*ACTG1* gene expression. Among the top eleven HFGs associated with DCM (Table 1), there were the three highly recurrent 3′-*ACTG1*-fused HFGs: *NDUFV1*-*ACTG1*, *TINAGL1-ACTG1*, and *TPM2-ACTG1* (Figure 2B). These three HFGs were detected in 72.9%, 50.9%, and 24.6% of left ventricle heart tissues of 122 DCM patients. On the other hand, they were detected in 17.9%, 22.2%, and 11.9% of left ventricle heart tissues of 252 GTEx healthy controls. The DCM *NDUFV1*-*ACTG1*, *TINAGL1-ACTG1*, and *TPM2-ACTG1* were 4.1-, 2.3-, and 2.1-folds of the GTEx healthy controls, showing that these highly recurrent 3′*ACTG1*-fused HFGs had less impact on DCM than other*3′ACTG1*-fused HFGs such as *TTN-ACTG1* and *PFKP-ACTG1*. The high recurrent frequencies of these 3′-*ACTG1*-fused HFGs in GTEx healthy controls supported that these HFGs were inherited from their parents, not from the random somatic genomic abnormalities. It confirmed the theoretical basis of our statistical approach to studying HFGs.

Similarly, we found twenty 3′*TTN*-fused HFGs associated with DCM, from 3.1-fold to 37.2-fold of the GTEx counterparts (Supplementary Table S4). Table 1 showed that two highly recurrent *TTN-LSM1* and *TTN-LMF1* HFGs of twenty-one 3′-*TTN*-fused HFGs were detected in 40.2% and 23.8% of 122 DCM patients and were 5.3- and 8.6-fold of the GTEx counterparts. Figure 2C showed that *TTN* and *LSM1* were located on 2q31.2 and 8q11.2 and encoded titin and LSM1 homolog. A potential translocation produced a *TTN-LSM1* fusion structure to generate *TTN-LSM1* HFG encoding 3664 aa titin- LSM1 homolog fusion protein, which was only 11% of 33,430 titin (NM_133378.4).

After performing a statistical analysis of HFGs between DCM and GTEx, we discovered four HFGs that were negatively associated with DCM. These six HFGs were detected in 0.8% to 36.1% of 122 DCM patients. In contrast, they were observed in 11.5% to 51.6% of 252 GTEx healthy controls. The latter were from 1.9 to 8.7 folds of the former, suggesting that these four HFGs reduced and retard DCM development and prognosis. The most significant difference between DCM and GTEx was *CCDC76-ARSA* HFG, detected in 0.8% of 122 DCM patients and 13.1% of 252 GTEx healthy controls. The most frequently observed HFG was *GPR82-POPDC2*, detected in 36.1% of DCM patients and 51.1% of GTEx healthy controls. The numbers and frequencies of HFGs positively associated with DCM were significantly higher than those of HFGs negatively associated with DCM, suggesting that these HFGs overwhelmingly led to DCM under the environmental conditions in which these DCM patients lived.

**FIGURE 3:**
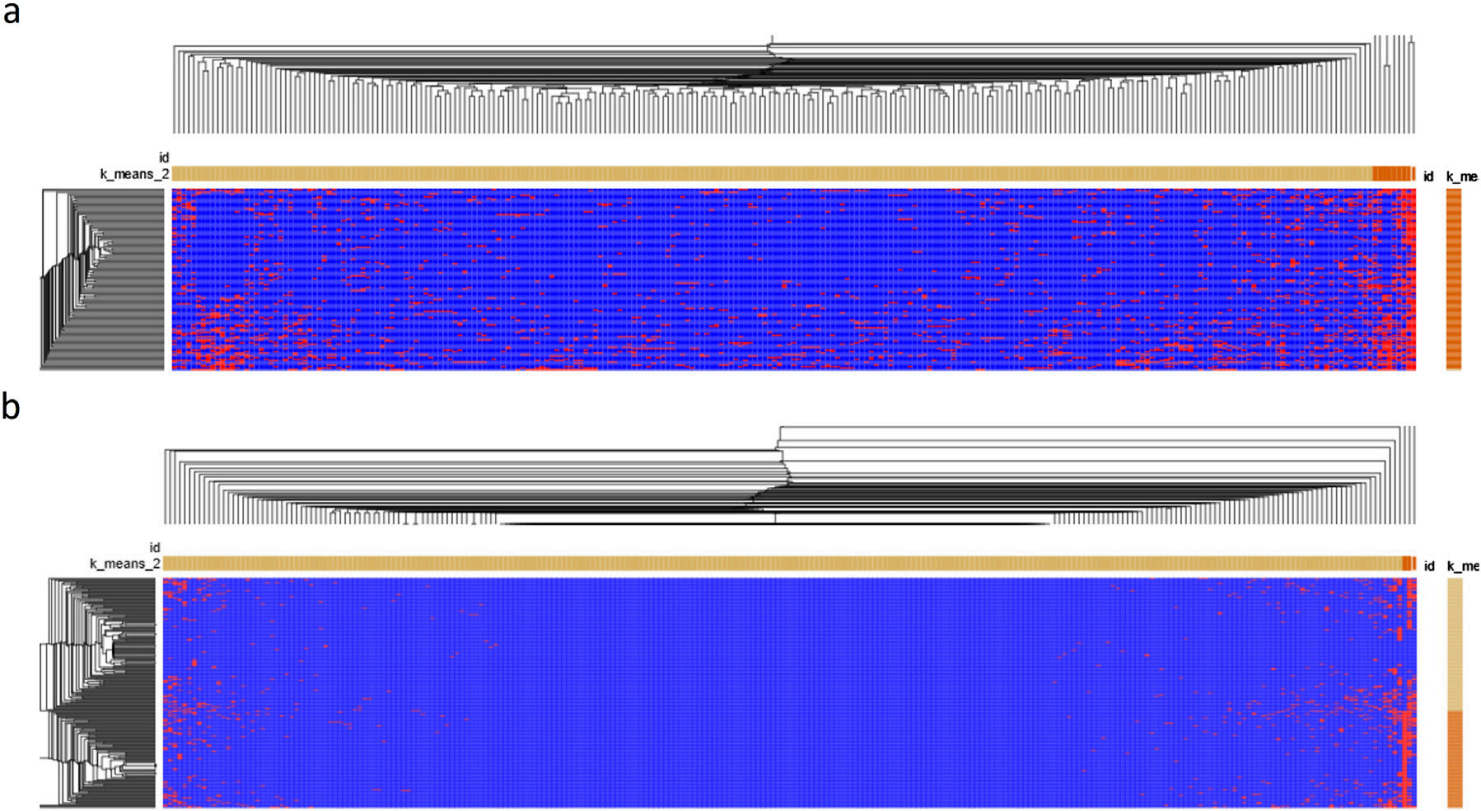
The heatmaps of HFG transcripts associated with DCM. **(A)** Morpheus generated a Germline HFG heatmap of 122 DCM patients from Broad Institute (https://software.broadinstitute.org/morpheus/). We used Morpheus to create a heatmap of 224 HFG transcripts of the 122 DCM patients. **(B)** Morpheus generated a heatmap of 224HFG transcripts of GTEx healthy controls from Broad Institute. K-means clustering was used to cluster rows and columns using Euclidean distance (number clustering of 2). Then, hierarchical clustering was used to cluster both rows and columns using Euclidean distance and grouping both rows and columns. The results were saved as a PDF document. The horizontal orange and light-yellow rectangles represented sparsely and highly recurrent HFG transcripts. The vertical orange and light-yellow rectangles represented HFGs-poor and HFG-rich DCM patients/GTEx healthy controls.

To show the relationship between HFGs and DCM patients, we used Morpheus (https://software.broadinstitute.org/morpheus/) to generate a heatmap of 224 HFG transcripts of 122 DCM patients. Figure 3A shows the heatmap of 224HFG transcripts associated with DCM among 122 DCM patients. Only twelve HFG transcripts were clustered into the highly recurrent HFG group and corresponded to the HFG transcripts in Table 1. The rest of the HFG transcripts were clustered into the sparsely recurrent group, and their distribution was not uniformly distributed, supporting that these HFGs played roles in DCM developments among 122 DCM patients. Figure 3B shows the heatmap of 224 HFG transcripts of GTEx healthy controls 224 HFG transcripts were clustered into highly recurrent and sparsely recurrent groups. Figure 3B indicates that the GTEx highly recurrent HFG transcripts had about five HFG transcripts, three of which were negatively associated with DCM. Most GTEx sparsely recurrent HFG transcripts were “empty” on the Figure 3B heatmap and were not frequently observed in GTEx healthy controls. A comparison between the heat maps in Figures 3A, B showed that DCM patients had quite different HFG signatures from those of GTEx.

### Identification of epigenetic fusion genes (EFGs) associated with DCM

**FIGURE 4:**
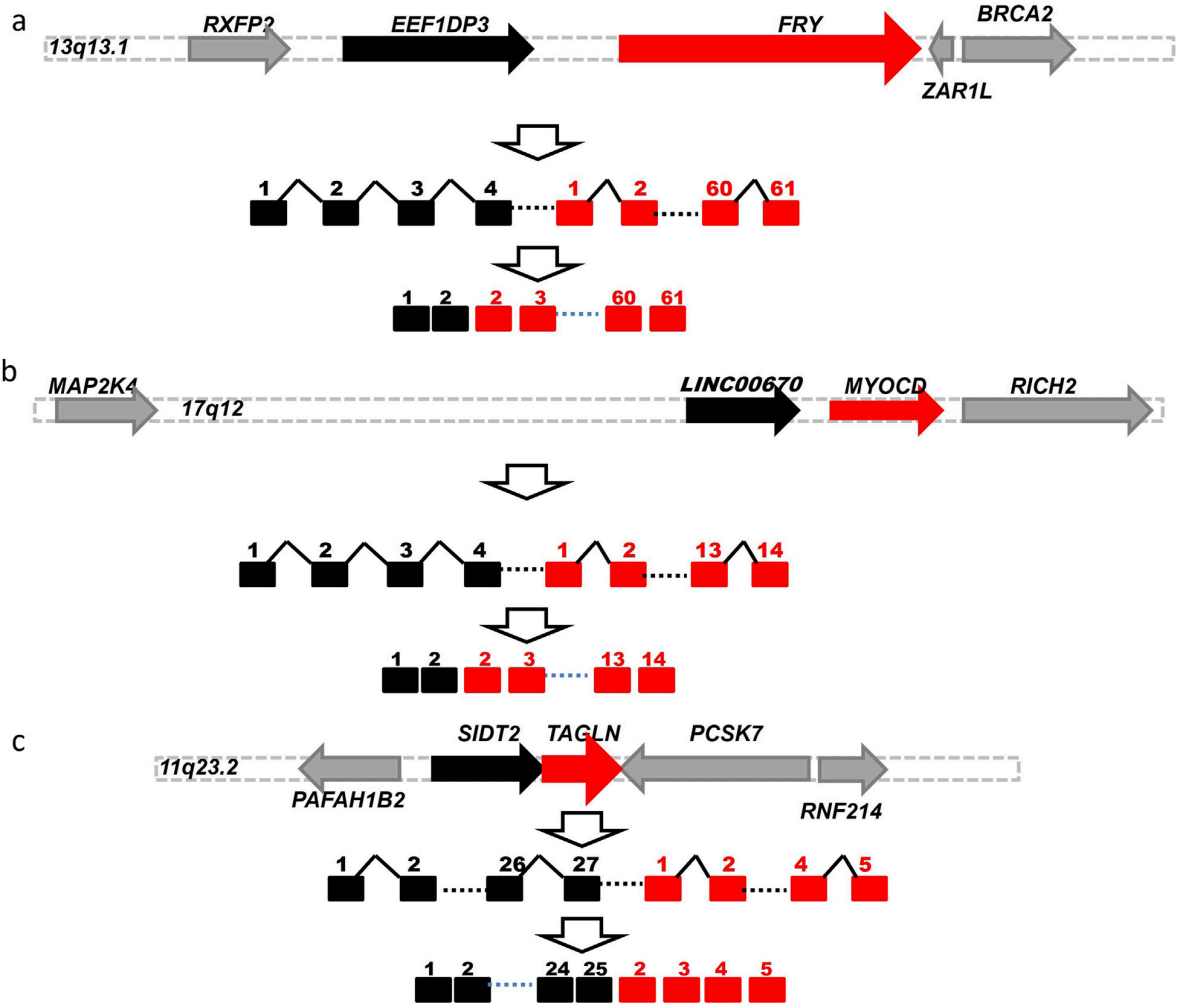
Schematic diagrams of potential genomic events to generate EFGs associated with DCM. Schematic diagrams showed potential mechanisms to generate *EEF1DP3-FRY* **(A)** *LINC00670-MYOCD* **(B)** and *SIDT2-TAGLN* **(C)** generated via *cis*-splicing of readthrough pre-mRNAs of two identical-strand neighboring genes. The solid black, red, and gray horizontal arrows represented the 5′genes, 3′gene, and genes surrounded by 5′and 3′genes. The solid black and red squares were 5′and 3′exons. The solid black angle line and dashed line were introns and omitted sequences. Open vertical arrows indicated potential steps from transcription readthrough to producing fusion gene sequences. The numbers above the squares were exon numbers.

As described above, we identified 181 EFGs encoding for 217 EFG transcripts positively associated with DCM, ranging from 4% to 99.2% of left ventricle heart tissues of 122 DCM patients. In contrast, the GTEx counterparts ranged from 0.4% to 81%. The former was from 1.2-fold to 237.5-fold of the latter. Table 2 showed 22 EFGs with recurrent frequencies of ≥50% of 122 DCM patients, ranging from 50% to 99.2%, while their GTEx counterparts ranged from 0.4% to 81%. The former were 1.2- to 237.5-fold of the latter. The most recurrent *EEF1DP3-FRY* EFG was detected in 99.2% of 122 DCM patients and 52.8% of 252 GTEx healthy controls. The former was 1.9-fold of the latter. Figure 4A shows that *EEF1DP3* and *FRY* were located on 13q13.1 and encoded eukaryotic translation elongation factor 1 delta pseudogene 3 and furry homolog (*Drosophila*). The *EEF1DP3* transcription termination failure resulted in *EEF1DP3-FRY* readthrough pre-mRNAs, which were spliced into *EEF1DP3-FRY* HFG and encoded a truncated 2963 aa furry protein (Figure 4A).

**TABLE 2:** The most highly-recurrent epigenetic fusion genes (EFGs) associated with DCM.

EFG ID	DCM (122)		GTEx (252)		Folds
	#	%	#	%	
*EEF1DP3-FRY*	121	99.2	133	52.8	1.9
*DCUN1D2-ADPRHL1*	120	98.4	105	41.7	2.4
*IGSF5-PCP4*	118	96.7	204	81.0	1.2
*CDKL3-SKP1*	114	93.4	100	39.7	2.4
*CKMT2-ZCCHC9*	108	88.5	100	39.7	2.2
*ZNF782-ZNF510*	105	86.1	75	29.8	2.9
*MRPS10-GUCA1B*	99	81.1	68	27.0	3.0
*TM9SF3-TLL2*	95	77.9	75	29.8	2.6
*MTG1-SCART1*	84	68.9	105	41.7	1.7
*SLC29A1-HSP90AB1*	80	65.6	76	30.2	2.2
*CTNNBIP1-CLSTN1*	78	63.9	42	16.7	3.8
*SCART1-CYP2E1*	73	59.8	104	41.3	1.4
*DNAJC25-GNG10-UGCG*	72	59.0	24	9.5	6.2
*PRSS42-PRSS45*	71	58.2	46	18.3	3.2
*PLEKHM1P-LOC146880*	68	55.7	88	34.9	1.6
*MARCH2-HNRNPM*	64	52.5	45	17.9	2.9
*AGGF1-LOC728723*	63	51.6	29	11.5	4.5
*SLC25A16-DNA2*	61	50.0	63	25.0	2.0

The EFG with the most significant difference between DCM and GTEx was *LINC00670-MYOCD*, detected in 23% of 122 DCM patients and 0.4% of 252 GTEx healthy controls. The former was 57.8-fold of the latter, suggesting that *LINC00670-MYOCD* was one of the most significant biomarkers associated with DCM. Figure 4B shows that *LINC00670* and *MYOCD* were located on 17q12 and encoded long intergenic non-protein coding RNA 670 and myocardin. Figure 4B showed that failed *LINC00670* transcription termination resulted in *LINC00670-MYOCD* readthrough pre-mRNAs, which were spliced into *LINC00670-MYOCD* EFG, coding for a truncated 907 myocardin, shorter of eighty-six aa than the native one. Among 486 EFG transcripts of ≥5 DCM patients, we identified only one *SIDT2-TAGLN*EFG negatively associated with DCM, detected in 23% of DCM patients and 40.5% of GTEx healthy controls. The latter was 1.8-fold of the former, suggesting that *SIDT2-TAGLN*EFG reduced and prevented DCM development and prognosis. Figure 4C showed that *SIDT2* and *TAGLN* were located on the 11q23.2 plus strand and formed a *SIDT2-TAGLN* EFG via cis-splicing of *SIDT2-TAGLN* readthrough pre-mRNAs. *SIDT2-TAGLN* putatively encoded a 1,038 aa SID1 transmembrane family member 2-transgelin fusion protein.

**FIGURE 5:**
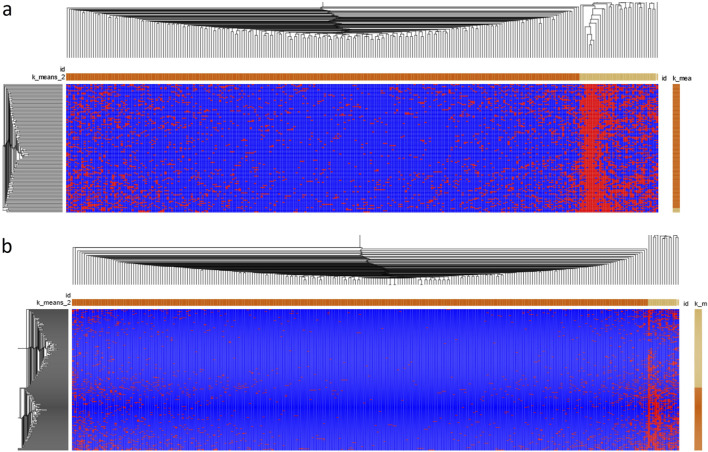
The heatmap of EFG transcripts associated with DCM. **(A)**. A heatmap of 181 EFGs encoding 221 EFG transcripts of 122 DCM patients was generated by Morpheus from Broad Institute (https://software.broadinstitute.org/morpheus/). We used Morpheus to create a heatmap of 221 EFG transcripts of the 122 DCM patients. **(B)** A heatmap of 221EFG transcripts of GTEx healthy controls was generated by Morpheus from Broad Institute. K-means clustering was used to cluster rows and columns using Euclidean distance (number clustering of 2). Then, hierarchical clustering was used to cluster both rows and columns using Euclidean distance and grouping both rows and columns. The results were saved as a PDF document. The horizontal orange and light-yellow rectangles represented sparsely and highly recurrent EFG transcripts. The vertical orange and light-yellow rectangles represented EFGs-poor and EFG-rich DCM patients/GTEx healthy controls.

We used Morpheus with identical conditions to analyze EFGs associated with DCM. Figure 5A shows the heatmap of 220 EFG transcripts among 122 patients. EFG transcripts were classified into highly recurrent and sparsely recurrent EFG transcripts, which were 13.3% and 86.7%. There were no dramatic differences in EFG transcripts among 122 DCM patients. These suggested that common environmental and genetic abnormalities resulted in DCM. Figure 5B shows that 220 EFG transcripts were distributed among 252 GTEx healthy controls. The 220 EFG transcripts were clustered into highly recurrent and sparsely recurrent groups. As described above, only *SIDT2-TAGLN* EFG was negatively associated with DCM, which was detected in 40.5% of GTEx healthy controls. Hence, the rest of the highly-recurrent EFG transcripts were highly recurrent in DCM patients and GTEx healthy controls. The differences between DCM patients and GTEx healthy controls were significant but small. Comparison between Figures 5A, B heatmaps shows that the DCM EFG signatures became wildly divergent from the GTEx ones and were consistent with increasing expressions of EFGs during the DCM development.

## Discussion

Previously, we used the splicingcode theory to develop a software system to discover fusion gene transcripts at maximum accuracy. To implement this algorithm of achieving the most accurate fusion transcripts, we used a splicingcode table counting for 0.5% of human genome sequences. In theory, we expected significantly fewer fusion transcripts to be identified than the software available so far (Zhou et al., 2017). 138,000 DCM and 49,900 GTEx fusion transcripts discovered in this study (Figure 1B) were much larger than those reported in the literature. Even though each DCM patient had three RNA-Seq samples, the difference between 855 and 814 fusion transcripts of ≥5 RNA-Seq SRA samples and ≥5 DCM patients (Figure 1C) was <5% and suggested that fusion transcripts of ≥5 samples were not three-fold theoretically and were the samples’ intrinsic characteristics. It also indicated that the ceilings of technologies used limited the number of highly recurrent fusion transcripts. Extra fusion transcripts were discovered by increasing RNA-Seq samples beyond the technological ceilings, behaved like random events, and were not essential to finding the samples’ valuable properties. On the other hand, much smaller numbers of fusion transcripts discovered by other software available made fusion transcripts like random events. Consequently, fusion genes were thought to be generated by random somatic genomic abnormalities associated with cancer.

Since highly recurrent fusion transcripts were unlikely generated by random genomic abnormalities in non-cancerous tissues, where somatic genomic rearrangements had been considered highly rare (Gottlieb et al., 2010), we assumed that if structural variants generating a fusion gene per individual had a rate of 3.6 × 10^−2 41,42^, five unique individuals possessing ≥5 identical fusion genes by identical genomic alterations were 6 × 10^−8^. The chances of discovering identical fusion genes of ≥5 samples in 122 DCM patients and 252 GTEx healthy controls would be far smaller than 7.4 × 10^−6^ in DCM patients and 1.5 × 10^−5^ in GTEx healthy controls. These low possibilities suggested that somatic genomic rearrangements did not generate these fusion genes simultaneously; instead, they were inherited from their parents (Zhuo, 2022). We used well-documented germline *KANSARL* (*KANSL1-ARL17A*) fusion gene specific to 29% of the population of European ancestry but absent in the populations from Asia and Africa (Zhou et al., 2017) as a reference. We used three sets of statistical parameters to compare *KANSARL* fusion gene among different populations and identify the statistical parameters to generate consistent results. We further used EFGs of different RNA-Seq datasets to validate if these parameters can generate the consistent results. After extensive manual and computational analysis, we set α = 0.01 (α/2 = 0.005), Z_α/2_ = 2.576, and n*p ≥ 5 and performed direct statistical analysis to fusion transcripts of ≥5 DCM patients between 122 DCM patients and 252 GTEx healthy controls. The identification of 224HFG transcripts among DCM patients and healthy controls supported the idea that they were inherited from their parents instead of somatic abnormalities.

Previously, we used the HFGs discovered in monozygotic twins (Zhuo, 2022) to study whether these HFGs were associated with multiple myeloma (Fei et al., 2022), acute myeloid leukemia (Ling and Zhuo, 2022), and amyotrophic lateral sclerosis (Yang et al., 2023). In this study, we have developed a statistical method to perform a comparative analysis of total fusion transcripts of 122 DCM patients and 252 GTEx healthy controls. We identified 210 HFGs encoding 224 fusion transcripts, which were statistically significant and ranged from 4.1% to 87.7%. Ninety-eight percent of 210 HFGs were positively associated with DCM development and prognosis. These 206 HFGs promoted DCM initiation, development, and progress. Only four HFGs were negatively associated with DCM and had preventive effects on DCM. Even though DCM patients and GTEx healthy controls were two different random populations, many factors may affect the correct identification of HFGs associated with DCM. RNA-Seq methods, RNA-Seq sequencing sizes, and insert sizes were the most important factor affecting the analysis accuracies. To overcome potential pitfalls, we must select the dominant isoforms of reference HFGs and EFGs to determine if DCM and GTEx samples behaved similarly. The second most important issue of using RNA-Seq to discover HFGs is that HFGs are differentially expressed. For example, many HFGs and EFGs were expressed in left ventricle heart tissue, not expressed in the blood samples. Therefore, we cannot use HFGs and EFGs discovered in blood samples to analyze RNA-Seq data of left ventricle heart tissue. The ages and races of the two populations would significantly affect the correct identification of HFGs associated with DCM. The sources of the sample collections and origins (e.g., car accidents vs. transplants) will greatly affect the correct HFG identification. Since healthcare services were localized, high recurrent HFGs of a local population may be due to genetic drift and were like local environmental factors affecting the identification of EFGs associated with DCM.

The most highly recurrent HFG was *RYR2-ACTN2*, detected in 107 (87.7%) out of 122 DCM patients and 54 (21.4%) out of 252 GTEx healthy controls. These large numbers of *RYR2-ACTN2* fusion genes in both DCM patients and GTEx healthy controls supported the fact that these highly recurrent HFG fusion genes were germline and confirmed the soundness of our statistical approach to fusion genes in non-cancerous tissues (Zhuo, 2022). 210 HFGs that were identified to be associated with DCM were much more than the numbers of “inherited” mutated genes, which were thought to count for 20%–40% of familial DCM cases (Mestroni et al., 1999), suggesting that “inherited” HFGs were dominant genetic factors associated with DCM and contributed 71.3% of total factors associated with DCM. In comparison, environmental abnormalities contributed to 28.7% of DCM. The low recurrent frequencies of the most “inherited” mutated genes in DCM patients (Chen et al., 2021) further supported that HFGs were more critical “inherited” genetic factors. Since mutated *TTN* was one of the most essential “inherited” genetic factors (Chen et al., 2021), identifying 20 3′*TTN*-fused HFGs confirmed that defected *TTN* was associated with DCM. However, it was essential to understand that the mutated *TTN* gene and 3′-*TTN-*fused HFGs were partially responsible for DCM. We must consider the complex developmental interactions between genetic and environmental abnormalities that lead to DCM initiation, development, and prognosis.

If a sample had one and/or more copies of RNA-Seq reads having fusion gene junction sequences of a fusion gene discovered by SCIF, this sample was thought to be fusion gene-positive (Zhou et al., 2017). Hence, enormous computation-assistant approaches exist to identify at least one of these RNA-Seq reads. We provided a validation algorithm using fusion junction sequences in Supplementary Tables S1, S2 to validate our analysis results (see Materials and Methods). This algorithm validated the most results except for some HFGs generated via highly repetitive genomic sequences. More importantly, this method identified HFGs and EFGs in other RNA-Seq datasets to study heart and diabetes diseases.

Our previous study showed that the total copy numbers of fusion gene isoforms were positively related to fusion gene expression levels36. Generally, the higher the sample numbers of fusion genes were positive, the easier the fusion genes were to be amplified by RT-PCR. Hence, one could use BLAST 5′ and 3′ fusion gene junction sequences of Supplementary Tables S1, S2 to experimentally validate fusion genes to locate the 5′ and 3′ exon sequences. Then, 5′ and 3′ exon sequences were merged into fusion gene sequences, based on which 5′ and 3′ RT-PCR primers were designed. This pair of primers was used for RT-PCR amplification of RNA sequences. Then, RT-PCR products were isolated and sequenced. If the RT-PCR products had sequences identical to the fusion junction sequence, we validated it as the fusion gene. We selected the top 21 fusion transcripts of the most highly recurrent fusion genes discovered in multiple myeloma. We validated all of them in multiple myeloma patients, confirming that highly recurrent HFGs and EFGs were reproducible and ready to be validated (In preparation). Since many DCM HFGs and EFGs were differentially expressed, we must consider the age and tissue types for easy validation.

As reported previously, EFGs reflected these developmental interactions between genetic and environmental abnormalities. Figure 5A shows that large numbers of EFG transcripts were classified together and highly recurrent, suggesting that these EFG transcripts reflected interactions between genetic and environmental abnormalities. As shown in Supplementary Table S2 and Table 2, we had identified 181 EFGs coding for 221 EFG transcripts. 180 EFGs of 181 EFGs were positively associated with DCM, and only one EFG was negatively associated with DCM. These overwhelming numbers of positively associated EFGs suggested that environmental conditions in which 122 DCM patients lived promoted DCM initiation, development, and progress. The differences in the highly recurrent EFGs between DCM patients and GTEx healthy controls in Table 2 were smaller than the HFG counterparts in Table 1. They confirmed that “inherited” HFG transcripts were much stronger forces of developing DCM than environmental factors. Environmental factors become powerful only in the presence of “inherited” genetic factors associated with DCM. If no genetic factors were associated with DCM, only persistent long-term environmental abnormalities might result in DCM initiation, development, and progress. In the future, we can genotype the general populations including children. Based on their highly recurrent HFG genotypes, we should provide personalized guides to individuals to increase physical activities to decrease possibilities to develop DCM by monitoring EFG genotypes. Consequently, we will require the identification of shared environmental factors to promote DCM and reduce these HFG and EFG interactions to minimize DCM initiation and development. Furthermore, we used medical and environmental interventions to interrupt and retard DCM initiation, developments, and progression initialized by HFGs.

## Data Availability

The data presented in the study are deposited in the Figshare repository, accession number 27096157: https://doi.org/10.6084/m9.figshare.27096157.v1.
